# Counteractive effects of predator invasion and habitat destruction on predator–prey systems

**DOI:** 10.1002/ece3.11646

**Published:** 2024-07-04

**Authors:** Jing Zhang, Linying Wang, Yinghui Yang, Haoqi Liu

**Affiliations:** ^1^ College of Mathematics and Computer Science Zhejiang Agriculture and Forestry University Hangzhou China; ^2^ School of Mathematics Southwest Jiaotong University Chengdu China

**Keywords:** community stability, habitat destruction, invasion control, invasive predators

## Abstract

Alien species invasion and habitat destruction are among the primary threats to native animal communities, particularly for native predator–prey systems. However, when predator invasion and habitat destruction co‐occur, it remains unclear whether their respective threats to native systems compensate each other or accumulate, as well as how these effects respond to the different characteristics of predator invasion and habitat destruction. In this study, we developed a spatially explicit simulation model with one prey species and one predator species and exposed it to invasive predators and habitat destruction with different properties. The results revealed the following insights: (1) Habitat destruction can compensate threats to native predator–prey systems from global predator invasion only when native predators possess predation capability similar to those of the invaders. In other scenarios, cumulative effects arise from predator invasion and habitat destruction. (2) Low levels of habitat destruction occurring at a faster rate, in conjunction with a substantial number of global invasive predators being present, can better compensate their respective threats to native predator–prey systems than the other scenarios. These findings provide valuable insights into situations where habitat destruction and alien species invasion coincide. They raise the question of whether we can leverage the interaction between them to reduce threats to biodiversity.

## Graphical abstract

Habitat destruction can compensate threats to native predator–prey systems from global predator invasion only when native predators possess predation capability similar to those of the invaders. Low levels of habitat destruction occurring at a faster rate, in conjunction with a substantial number of global invasive predators being present, can better compensate their respective threats to native predator–prey systems than the other scenarios.

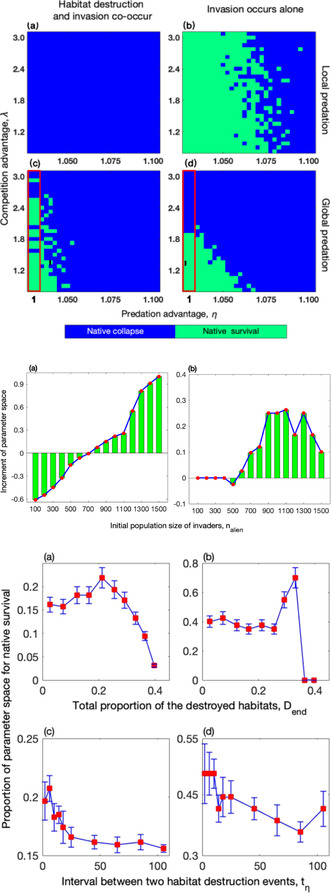

## 1 INTRODUCTION

Biological invasions pose a serious threat to global biodiversity (Clarke & McGeoch, 2023; Enders et al., 2018; Pysek et al., 2020). When alien species are introduced to resident communities, some may strongly compete with native species due to niche overlap (Hänfling et al., 2011; Maron & Marler, 2008). Additionally, certain alien species can become invasive predators by preying on native species, such as cats (*Felis catus*), rats (*Rattus rattus*), stoats (*Mustela erminea*), and mongooses (*Herpestes auropunctatus*) (Cirovic et al., 2011; Doherty & Ritchie, 2016; Jones et al., 2011). The presence of these invasive predators can disrupt native food webs, alter ecosystem processes, and diminish local biodiversity (Gregory et al., 2024; Wardle et al., 2011). Consequently, mitigating the negative impacts of invasive species, especially invasive predators, on native food webs has become a primary objective of conservation agencies worldwide (Doherty & Ritchie, 2016; Márquez et al., 2024).

Habitat destruction is another significant threat to food webs (Crooks et al., 2017). The loss, fragmentation, and alteration of natural or semi‐natural habitats have led to ecosystem destabilization, loss of crucial resources, and genetic and cultural impoverishment (Hazard et al., 2023; Ribas et al., 2005). For instance, habitat loss due to the construction of a hydroelectric dam in the Brazilian Amazonia led to ecosystem destabilization, resulting in an abrupt disruption of terrestrial predator–prey networks and the loss of functionality (Pires et al., 2023). Furthermore, habitat loss has intensified over the past two centuries without signs of slowing (Dapporto & Dennis, 2008; Schlosser & Pfirman, 2012). Therefore, ensuring the long‐term persistence of food webs in the face of habitat destruction is highly important (Ryser et al., 2019).

Previous studies have suggested that when biological invasions and habitat destruction occur together, these two factors can interact with each other (Gregory et al., 2024; With, 2004). It is widely believed that habitat destruction strongly affects invasive species. The current literature indicates that habitat destruction can promote the success of invasive species (Gallé et al., 2023; Kumschick et al., 2015). For example, With (2004) and Pearson and Dawson (2005) suggested that exotic species with greater dispersal ability may benefit more from habitat destruction. Recent field surveys of the American bullfrog *Lithobates catesbeianus* have shown that habitat destruction has facilitated the expansion and colonization of invaders within habitats with limited resources (Wang et al., 2021). However, the influence of habitat destruction on invasion is not universal across all sites. In central Texas, habitat destruction has been found to impede the spread of invaders with limited dispersal ability (Alofs & Fowler, 2010). Empirical field studies and a metapopulation model constructed by Liu et al. (2012) suggest that under conditions of competition pressure and habitat destruction, the spread of invasive species could be thwarted. Other theoretical models proposed by Brown et al. (2012), Barron et al. (2020), and Bozzuto et al. (2021) have also revealed the potential for habitat destruction to restrain the propagation of invasive populations.

Although the interaction between biological invasion and habitat destruction has garnered much attention (Alofs & Fowler, 2010; Gallé et al., 2023), understanding how these invasions and destruction collectively impact the long‐term persistence of native communities remains challenging. For instance, when habitat destruction and invasion co‐occur, native species have a greater chance of persistence than when invasion occurs on its own, particularly if invasive species possess stronger competition or dispersal ability than native species (Yang & Liu, 2023). However, the responses of more complex native communities to co‐occurring habitat destruction and invasion still require further investigation. Moreover, solely considering habitat destruction based on a single factor (e.g., quantity levels) is inadequate due to the complexity of its influence on communities. For example, random landscape destruction may lead to a greater decline in species richness than the loss of area through spatial aggregation (Yin et al., 2021). Furthermore, habitat destruction is a dynamic process influenced by multiple factors, such as destruction intensity, extent, frequency, and timing (Didham et al., 2012; Roxburgh et al., 2004; Rybicki et al., 2020), and should generally be considered a combined consequence of these factors.

To examine the persistence of native food webs in the face of co‐occurring biological invasion and habitat destruction, we aimed to investigate two pivotal questions: (1) Do the threats of alien species invasion and habitat destruction to native predator–prey systems have cumulative or counteractive effects? (2) Is this counteractive or counteractive effect influenced by the specific attributes of invasion and habitat destruction? Taking cues from other models (Jiabu & Li, 2023), we developed an individual‐based, spatially explicit simulation model initially comprising a native predator–prey system. Subsequently, we introduced a third species, invasive predators, to establish an invasive predator system. Within this framework, we assumed that native predator species could engage in prey hunting and reproduction using two predation strategies: local predation (predation in adjacent habitats) and global predation (predation across the entire landscape). Invasive predator species were assumed to adopt the same predation strategies as native predators but possess enhanced predation and competition capacity. Prey species were assumed to reproduce exclusively in adjacent habitats.

Next, we constructed a random habitat destruction model in which habitats were randomly subjected to destruction. We facilitated the evolution of the invasive predator system within the landscape according to the rules specified in the predator invasion model. Simultaneously, we employed habitat destruction models as a control group. Specifically, intact habitats were converted to destroyed habitats, and the invasive predator system was allowed to evolve within the altered landscape, also adhering to the rules outlined in the predator invasion model. This process was iterated, and subsequently, we compared results from both scenarios to address the first inquiry concerning cumulative versus counteractive effects. Furthermore, we systematically varied the characteristics of predator invasion or habitat destruction to explore the optimal conditions for the native predator–prey system under the concurrent occurrence of habitat destruction and predator invasion, thereby addressing the second inquiry regarding the relationship between counteractive or counteractive effects and invasion/habitat destruction characteristics. This study sheds light on whether habitat destruction promotes the resistance of native predator–prey system to invasive predators or accelerates the collapse of the native system.

## 2 MODELS AND METHODS

**FIGURE 1:**
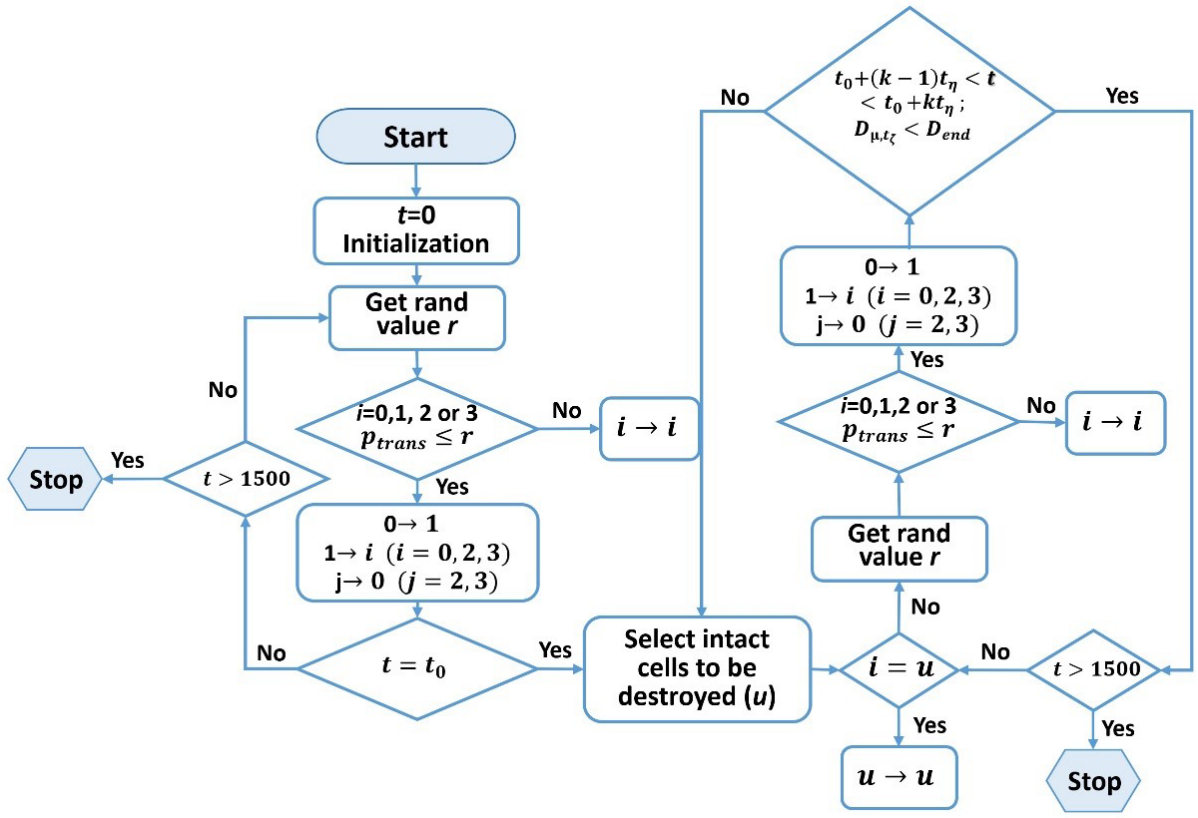
Flowchart of the model design and timing.

We developed an individual‐based and spatially explicit mechanistic model that simulated the stochastic dynamics of individuals on a lattice using cellular automata models (Wolfram, 1986). Initially, we simulated a native predator–prey system consisting of two species. Species 1 and 2 represented the native prey and predator, respectively. Once the native system reached a stable state, we introduced a third species and habitat destruction events. Species 3 represented an invasive predator with the same predation strategy as native species 2. We then further explored the population size of these species. The model design and timing are shown in Figure 1.

### 2.1 Predator–prey system model

We utilized a two‐dimensional lattice with 100 × 100 cells. The periodic boundary conditions, wherein individuals reappear on the opposite side of the system upon crossing a boundary, effectively mitigate the impact of boundary effects on the results. Therefore, this condition was implemented in the lattice. Each cell could be in one of four states: empty (0), occupied by native prey (1), native predator (2), invasive predator (3), or destroyed (4). Initially, native prey occupied 60% of the lattice, native predators accounted for 20% of the cells, and the remaining cells were empty. The distribution of these native species and empty cells was randomized across the lattice. Once the native prey and predators reached equilibrium, we introduced $n_{\text{alien}}$ alien predators into randomly selected empty cells.

The change in the state of each cell was determined by specific updating rules, which are listed in Tables 1 and 2 (Jiabu & Li, 2023). Regarding births, vacant sites were occupied by new offspring of native prey (1) from their nearest four neighbors. The colonization rate of native prey 1 was denoted as *b*. We assumed that the probability of a transition from state 0 to 1 was still proportional to the number of individuals surrounding species 1, represented by $p_{1}$. Hence, the transition rate of a cell from empty to state 1 would be $bp_{1}$, while the probability of an empty cell remaining empty would be $1-bp_{1}$. Individuals died due to natural mortality at a rate of $m_{i}$ (*i* = 1, 2, 3) (Hiebeler et al., 2016). Additionally, predators 2 and 3 might also die as a result of competition with each other. The parameter $c_{\mathrm{ij}}$ quantified the additional deaths caused by interspecific competition, with higher values indicating more intense competition. Thus, a cell in state 2 or 3 transitioned to an empty state 0 with a probability of $m_{i}+c_{\mathrm{ji}}p_{j}$ (where i,j=2,3 and j≠i). Consequently, the cell remained in the same state with a probability of $1-\left(m_{i}+c_{\mathrm{ji}}p_{j}\right)$ (where i,j=2,3 and j≠i). Regarding predation, we assumed that predators employ two predation strategies: local (refer to Table 1), which involves limited predation capability and interactions only with their immediate neighbors (using the von Neumann neighborhood with z=4), and global (refer to Table 2). Native prey 1 could be hunted by local predators 2 and 3 with probability $\alpha_{21}p_{2}$ and $\alpha_{31}p_{3}$, respectively. Under the global predation strategy, species 2 and 3 hunted for prey at rates of $\beta_{21}p_{2}^{g}$ and $\beta_{31}p_{3}^{g}$, respectively. All the parameters are comprehensively described in Table 3.

**TABLE 1:** Probabilities of local predation transitions from states along the rows to states along the columns.

	Empty 0	Species 1	Species 2	Species 3
Empty 0	$1-bp_{1}$	$bp_{1}$	—	—
Species 1	$m_{1}$	$1-m_{1}-\alpha_{21}p_{2}-\alpha_{31}p_{3}$	$\alpha_{21}p_{2}$	$\alpha_{31}p_{3}$
Species 2	${∆}_{1}≔m_{2}+c_{32}p_{3}$	—	$1-{∆}_{1}$	—
Species 3	${∆}_{2}≔m_{3}+c_{23}p_{2}$	—	—	$1-{∆}_{2}$

*Note*: $p_{i}$ (i=1,2,3) represents the proportion of species *i* among the nearest z=4 neighbors.

**TABLE 2:** Probabilities of global predation transitions from states along the rows to states along the columns.

	Empty 0	Species 1	Species 2	Species 3
Empty 0	$1-bp_{1}$	$bp_{1}$	—	—
Species 1	$m_{1}$	$1-m_{1}-\beta_{21}p_{2}^{g}-\beta_{31}p_{3}^{g}$	$\beta_{21}p_{2}^{g}$	$\beta_{31}p_{3}^{g}$
Species 2	${∆}_{1}≔m_{2}+c_{32}p_{3}$	—	$1-{∆}_{1}$	—
Species 3	${∆}_{2}≔m_{3}+c_{23}p_{2}$	—	—	$1-{∆}_{2}$

*Note*: $p_{i}$ (i=1,2,3) represents the proportion of species *i* among the nearest z=4 neighbors, and $p_{i}^{g}$ (i=2,3) represents the proportion of species *i* in the entire landscape.

**TABLE 3:** Parameters.

Parameter	Description	Value
$m_{i}$	Mortality rate of species *i* (i=1, 2,3)	0.2
b	Colonization rate of native species 1	
α or ηα	Local predation rate of species 2 (or 3) on species 1	
β or ηβ	Global predation rate of species 2 (or 3) on species 1	
η	Predation advantage of alien species 3 over species 2	
$c_{23}$	Interspecific competitiveness from species 2 to species 3	0.15
$c_{32}$	Interspecific competitiveness from species 3 to species 2	$\lambda c_{23}$
λ	Competition advantage of alien species 3 over species 2	
$n_{\text{alien}}$	Number of individuals for the invasive predators	
$D_{\mu ,t_{\zeta }}$	Proportion of destroyed habitats at step $t_{\zeta }$ for random habitat destruction	
$D_{\mathrm{end}}$	Total proportion of destroyed habitats	
$x_{\mu }$	Probability that each intact habitat becomes destroyed at each step for random habitat destruction	0.025
$t_{0}$	Time of introduction of habitat destruction	
$t_{\eta }$	Time interval between two habitat destruction events	

### 2.2 Habitat destruction model

**FIGURE 2:**
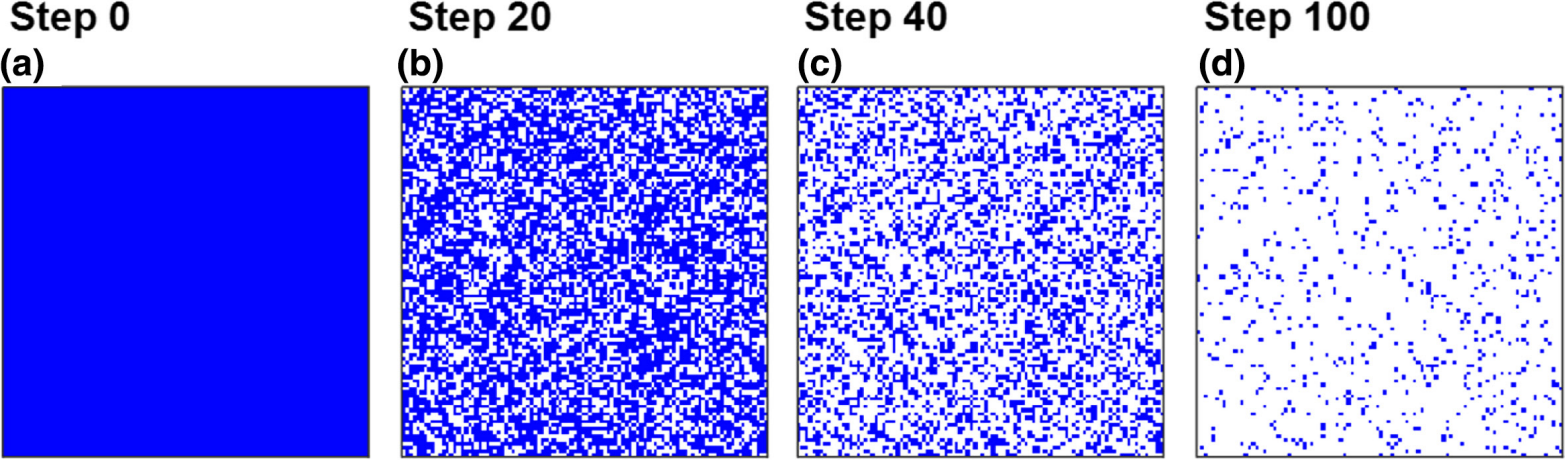
Examples of random habitat destruction processes. The blue areas are intact cells, and the white areas are destroyed cells. Parameters: $x_{\mu }=0.025$.

For the habitat destruction model, we constructed discrete models in both time and space, specifically random habitat destruction models. To develop the discrete model, the continuous landscape was partitioned into 100 × 100 habitats of equal area and shape. Initially, all the habitats were intact, and if destruction occurred in an intact habitat, this habitat was considered lost. The models were defined as follows: at each time step, each intact habitat had an equal probability $x_{\mu }$ (the value is presented in Figure 2) of being destroyed and becoming a lost habitat. We denote the proportion of destroyed cells at step $t_{\zeta }$ (where $t_{\zeta }=0,1,2,\ldots$) as $D_{\mu ,t_{\zeta }}$, which could be calculated as follows:

(1) $$D_{\mu ,t_{\zeta }}=1-{\left(1-x_{\mu }\right)}^{t_{\zeta }},D_{\mu ,0}=0.$$

### 2.3 Implementation method and data processing

We established parameter values for both the predator–prey system model and habitat destruction model (the values are shown in Table 3). Additionally, values were set for the total proportion of destroyed habitat ($D_{\mathrm{end}}$), the time of introduction of habitat destruction ($t_{0}$), and the time interval between two habitat destruction events ($t_{\eta }$) (values presented in the figures in the “Results” section). Initially, native prey and predators accounted for up to 60% and 20%, respectively, of the cells, while the remaining cells were empty. The spatial distribution of these native species was random. The simulations of invasive and native species dynamics and habitat destruction were implemented as follows:

1. We allowed the native species populations to dynamically change based on the predator–prey system model described in Section 2.1.
2. Once the native system reached stable, we selected $n_{\text{alien}}$ invasive predators and placed them randomly into empty cells. If habitat destruction was introduced, we initiated the first habitat destruction event at step $t_{0}$ using our habitat destruction procedure (refer to Section 2.2). If an intact habitat was destroyed, it could not be occupied by invaders or natives, and individuals living within it immediately died. We then allowed invader and native populations to dynamically change throughout the landscape, as described in Section 2.1, for the duration $t_{\eta }$. This process was repeated until the proportion of destroyed habitats reached $D_{\mathrm{end}}$. The landscape remained static, and we continued to allow all the species populations to dynamically change until the entire system reached equilibrium.
3. If habitat destruction was not introduced, we allowed the invasive and native populations to dynamically change throughout the landscape until the entire system reached equilibrium.

In all the simulations, there were no significant changes in the native species when simulation step 500 was reached before the introduction of the invasive species. After the introduction of invasive species and habitat destruction, no species exhibited significant changes once simulation step 2000 was reached. Therefore, we considered the native system to have reached equilibrium at step 500 before the introduction of invaders and considered the entire system to have reached equilibrium at step 2000. To eliminate stochasticity, ten independent replicate simulations were performed, and the figures in “Results” section were generated based on the 10 replicates. The parameter space where native prey and predator densities were positive in the equilibrium state for at least 6 replicates was considered the native predator–prey system survival region. The remaining parameter value combinations were considered to indicate native predator–prey system collapse. The proportion of parameter space in which the native system survives when habitat destruction and invasion co‐occur was defined by the ratio of the parameter space in which the native predator–prey system survives when habitat destruction and invasion co‐occur in at least 6 replicates relative to the entire parameter space. The increment in parameter space in which the native systems survive when invasion and habitat destruction co‐occur was calculated as follows: first, let the proportion of parameter space (denoted by $q_{1}$) in which the native systems survive when invasion and habitat destruction co‐occur minus the proportion of parameter space (denoted by $q_{2}$) in which only invasion occurs and obtain $q_{3}$; then, calculated the ratio of $q_{3}$ to $q_{2}$.

## 3 RESULTS

First, we investigated the long‐term persistence of the native predator–prey system when facing both predator invasion and habitat destruction concurrently in Section 3.1. Subsequently, we analyzed the attributes of invasive predators (Section 3.2) and habitat destruction (Section 3.3) that forecast the counteractive effects of invasion and habitat destruction on the indigenous predator–prey system.

### 3.1 Counteractive impacts of habitat destruction on global predator invasion

**FIGURE 3:**
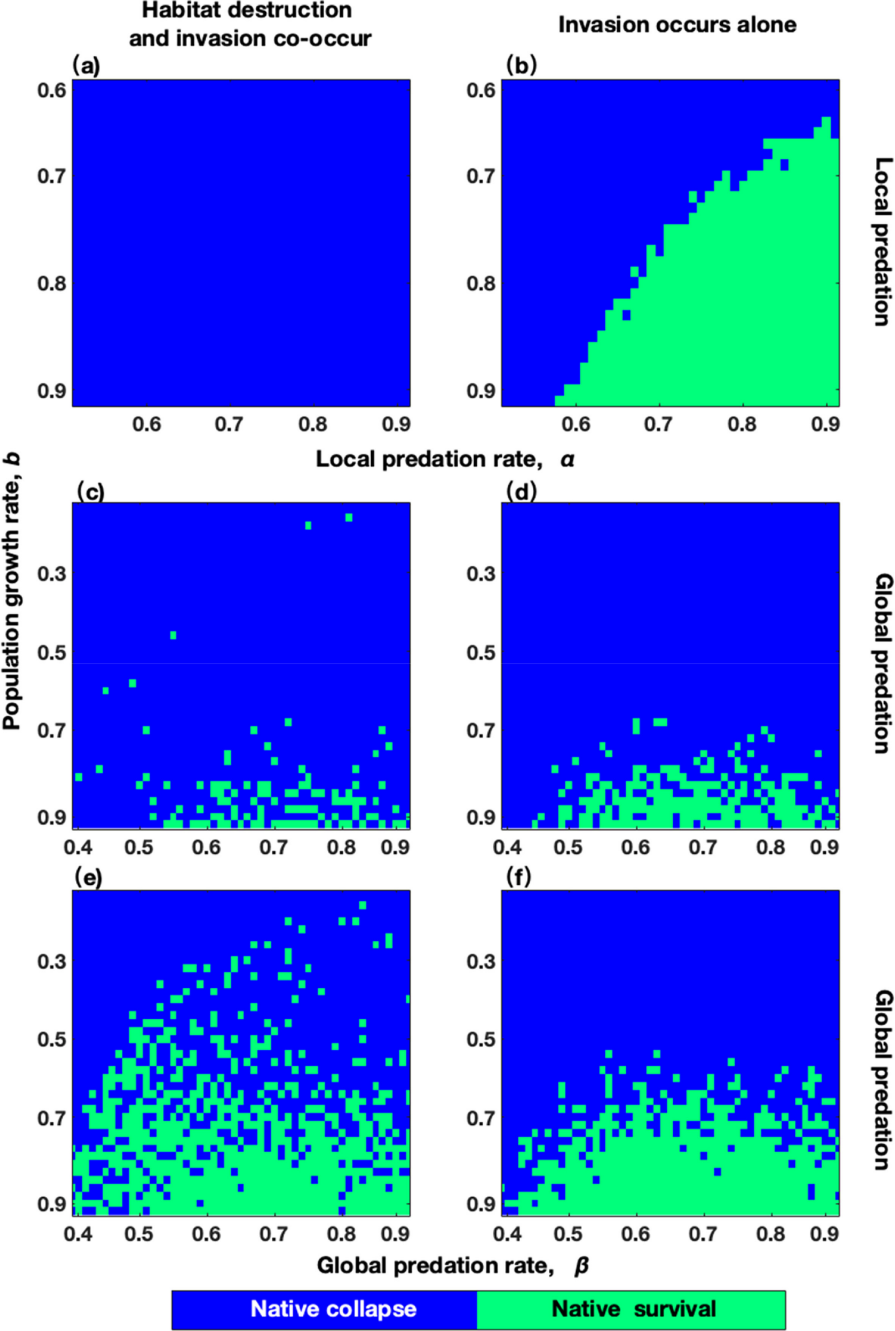
Habitat destruction aids native systems in resisting global invasive predators. Panels (a–f) display the parameter space (*b* and α for (a, b) and *b* and β for (c–f)) for the persistence of native systems when invaders possess competition advantages over the native (λ=1.67 for (a–f)) but invaders and natives have comparable predation ability (*η* = 1 for (a, b) and (e, f)) or when the invaders hold predation advantages over the natives (η=1.01 for (c, d)). The key parameters include $m_{i}=0.2$, $c_{23}=0.15$, $n_{\text{alien}}=1000$, $D_{\mathrm{end}}=21\%$, $t_{0}=\text{step}\;6$, $t_{\eta }=6$ steps, and $x_{\mu }=0.025$.

Figure 3b illustrates that the native system can still survive if the population growth rate of native prey (*b*) or the local predation rate of all predators (α) is greater than approximately 0.7 under predator invasion, when predators can engage in predation only locally. However, if habitat destruction and predator invasion occur simultaneously, the native system will likely collapse (Figure 3a). When predators prey globally, the parameter space (*b* and β, global predation rate of all predators) for the persistence of the native system under the co–occurrence of habitat destruction and predator invasion (green areas in Figure 3c) is smaller than that under invasion alone (green areas in Figure 3d) if the alien predators have a predation advantage over the native predators (η>1). The parameter space (*b* and β) for the persistence of the native system under the co–occurrence of habitat destruction and global predator invasion (green areas in Figure 3e) is larger than that under invasion alone (green areas in Figure 3f) only when native predators have predation capability similar to those of alien predators (η=1).

**FIGURE 4:**
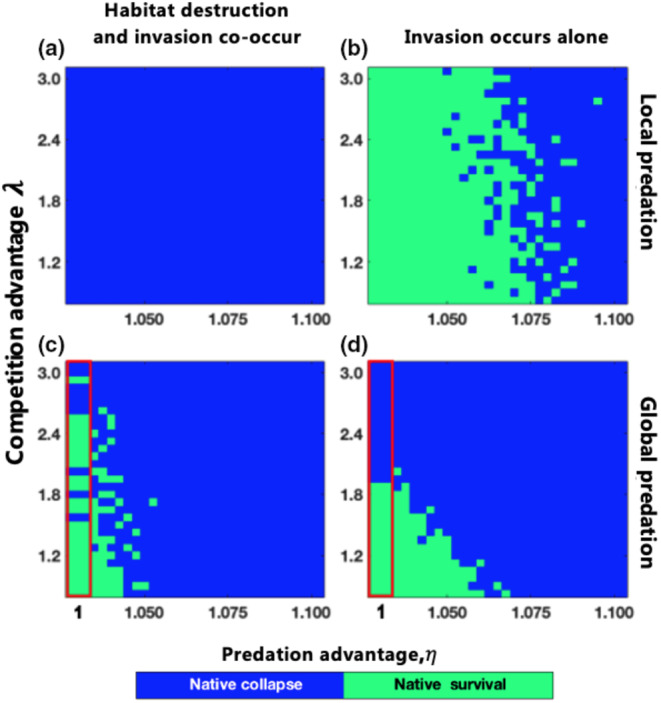
Habitat destruction aids native systems with competitive disadvantages in resisting global invasive predators. Panels (a–d) depict the parameter space (λ and η) for native system persistence when habitat destruction and invasion co‐occur or invasion occurs alone. The horizontal axis for the red region in panels (c, d) is labeled as 1. The parameters are as follows: (a, b) α=0.7; (c, d) β=0.6; b=0.8, $m_{i}=0.2$, $c_{23}=0.15$, $n_{\text{alien}}=1000$, $D_{\mathrm{end}}=29\%$, $t_{0}=\text{step}\;2$, $t_{\eta }=2$ steps, and $x_{\mu }=0.025$ for all panels.

Figure 4 illustrates that for global invasive predators, the parameter space (λ and η, competition and predation advantages of alien predators over native predators) for native system persistence under the co–occurrence of habitat destruction and invasion (green points in the leftmost column of Figure 4c) is larger than that under only invasion (green points in the leftmost column of Figure 4d), only when η=1 (the red region in Figure 4c,d). If the predator can only prey locally, the native system always collapses after the co–occurrence of habitat destruction and invasion (Figure 4a,b). In summary, when native predators present global predation similar to invasive predators and habitat destruction and invasion co–occur, native systems are more likely to survive than when invasion occurs alone; i.e., the threats of global predation capability invasion and habitat destruction may have a counteractive effect on the native system. Otherwise, habitat destruction always accelerates the collapse of the native system under predator invasion.

### 3.2 Counteractive interactions occur when facing numerous alien global predators

**FIGURE 5:**
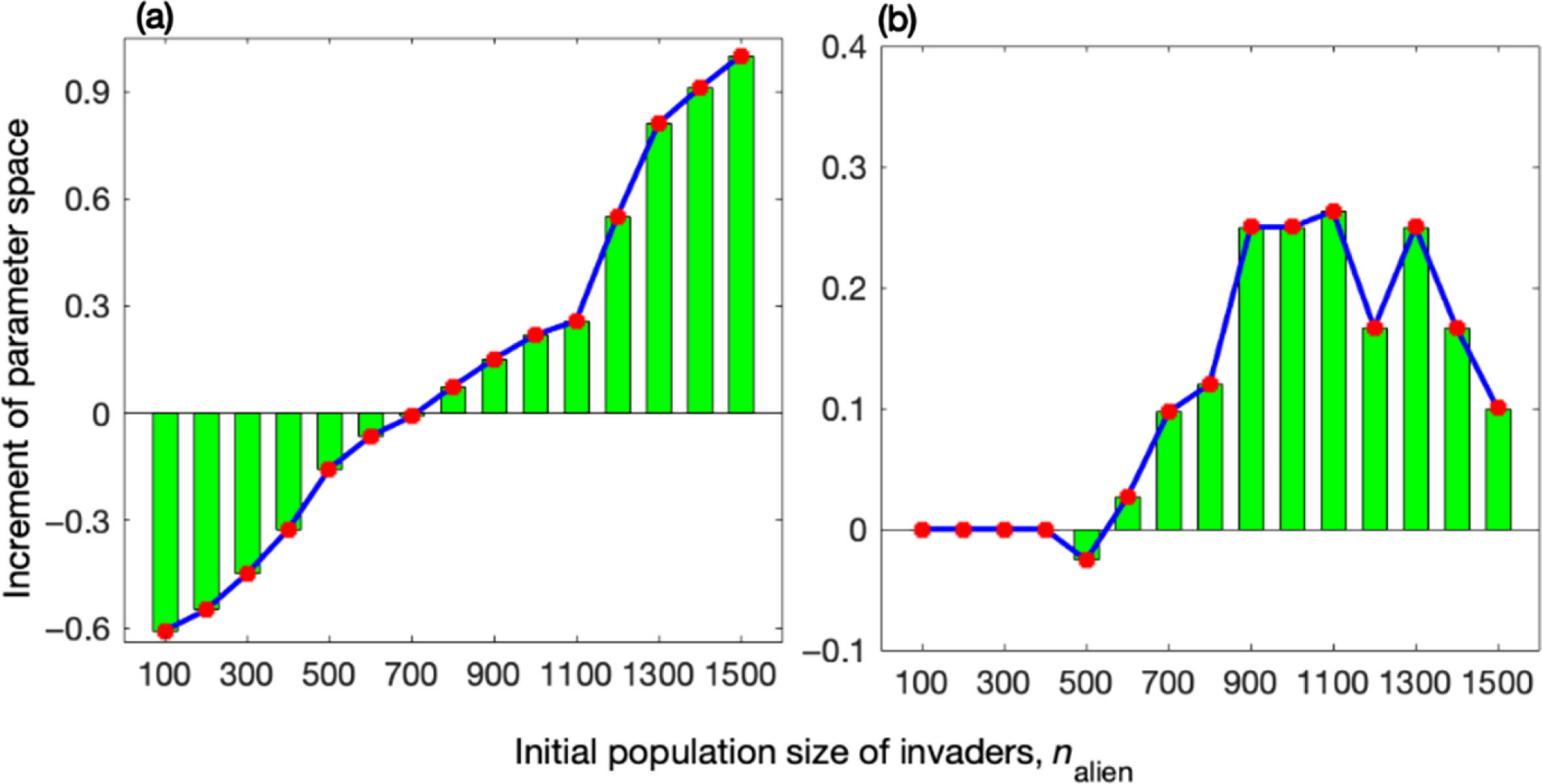
Habitat destruction enhances the persistence of native systems primarily in the presence of a substantial number of global invasive predators. Panels (a, b) showcase the correlations between the initial population size of global invasive predators ($n_{\text{alien}}$) and the increment of parameter space (*b* and β for (a) and λ and η for (b)) for the persistence of the native system when global predator invasion and habitat destruction co‐occur compared to the scenario where only invasion occurs (as defined in Section 2.3). For systems without habitat destruction, the parameters are set as follows: in panel (a), b=0.8 and β=0.6, while in panel (b), λ=1.67 and η=1. When considering both scenarios with and without habitat destruction, the parameters are set as follows: λ=1.67, η=1, $D_{\mathrm{end}}=21\%$, $t_{0}=\text{step}\;6$, and $t_{\eta }=6\;\text{steps}$ for panel (a); and b=0.8, β=0.6, $D_{\mathrm{end}}=29\%$, $t_{0}=\text{step}\;2$, and $t_{\eta }=2\;\text{steps}$ for panel (b). The parameters $m_{i}=0.2$, $c_{23}=0.15$, $n_{\text{alien}}=1000$, and $x_{\mu }=0.025$ apply to all panels.

In this section, we explored how the characteristics of invaders influence the counteractive effects of global invasive predators and habitat destruction on the native system. A breakpoint for the initial population size of alien global predators, $n_{\text{alien}}$, was observed at 700 (Figure 5a). When the value is lower than this threshold, the increase in the parameter space (*b* and β) for the persistence of native system when invasion and habitat destruction co‐occur, compared to when only invasion occurs, is negative. Conversely, when the value exceeds this threshold, the increase is positive and increases rapidly with increasing number of invaders. Similarly, in Figure 5b, a breakpoint is observed at 600, and above this breakpoint, the increase in the parameter space (λ and η) is also positive (Figure 5b); otherwise, the increase is negative. Therefore, it can be concluded that the parameter space for the persistence of native system when invasion and habitat destruction co‐occur is larger than that when only invasion occurs only when a significant number of alien global predators are present. Otherwise, the parameter space is smaller than that when only invasion occurs. In other words, habitat destruction and global predator invasion have counteractive effects on the native system when facing numerous alien global predators, whereas cumulative effects arise from predator invasion and habitat destruction in other scenarios.

### 3.3 Counteraction is stronger for low levels of habitat destruction

**FIGURE 6:**
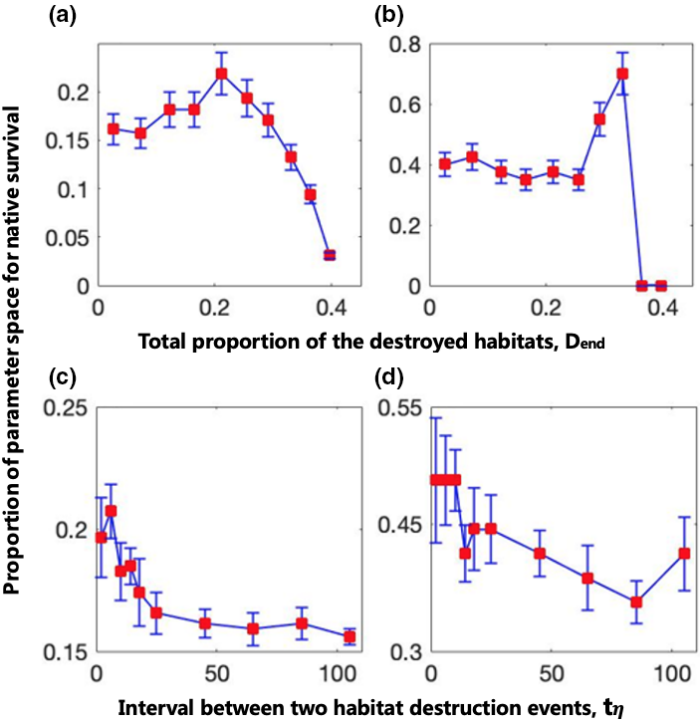
Low levels of habitat destruction occurring at a faster rate are more effective at enabling native systems to resist global invasive predators. Panels (a–d) show the relationships between the proportion of parameter space (b and β for (a) and (c), λ and η for (b) and (d)) in which the native systems can survive when habitat destruction and global predator invasion co–occur and the total proportion of the destroyed habitats, $D_{\mathrm{end}}$ (a, b), or the time interval between two habitat destruction events (refer to Section 2.3 for a definition), $t_{\eta }$ (c, d). The parameters are as follows: (a) $t_{\eta }=6\;\text{steps}$; (b) $t_{\eta }=2\;\text{steps}$; (c) $D_{\mathrm{end}}=21\%$; (d) $D_{\mathrm{end}}=29\%$; (a) and (c) λ=1.67, η=1, $t_{0}=\text{step}\;6$; (b) and (d) b=0.8, β=0.6, $t_{0}=\text{step}\;2$; $m_{i}=0.2$, $c_{23}=0.15$, $n_{\text{alien}}=1000$, and $x_{\mu }=0.025$ for all panels.

In this section, we investigated how the characteristics of habitat destruction affect the counteractive effects of global invasive predators and habitat destruction on the native system. The proportion of parameter space (*b* and β) for the persistence of the native system facing global invasive predators is highest (approximately 0.23 or 0.21 for Figure 6a,c) when the total proportion of destroyed habitats, $D_{\mathrm{end}}$, is approximately 0.21 (very low) or the time interval between two habitat destruction events, $t_{\eta }$, is approximately 4 (very short). Similarly, the proportion of parameter space for λ and η is highest (approximately 0.7 or 0.47 for Figure 6b,d) when $D_{\mathrm{end}}$ is approximately 0.33 (very low) or $t_{\eta }$ is approximately 4 (very short). Therefore, if the time interval between two habitat destruction events is very short (habitat destruction occurs at a faster rate) or if the level of habitat destruction is low, the native system has a greater chance of persistence when habitat destruction and global predator invasion co‐occur. In other words, habitat destruction and global predator invasion can more effectively compensate their respective threats to the native system.

### 3.4 Robustness of results

In this section, we conducted tests to assess the robustness of our results concerning variations in the parameters of the predator–prey model and habitat destruction model. Since the initial spatial distribution of invasive predators and the distribution of destroyed habitats significantly impact the outcomes, we examined the robustness of our findings by altering these two parameters. In our main results, we introduced invasive predators by randomly selecting empty cells. To test the robustness of the results, we employed a nonrandom spatial distribution of invasive predators known as “centre invasion,” where these were specifically placed in the center of the landscape (refer to Figure S1 in the Appendix S1 for details). Additionally, in contrast to the randomly distributed destroyed habitats used in the main results, we introduced clustered spatial distributions to assess robustness, particularly focusing on contagious habitat destruction (refer to Figure S2 in the Appendix S1 for details). We compared the results obtained from models with randomly distributed invasive predators and destroyed habitats (referred to as “models with original parameters”) to those from models with nonrandomly distributed invasive predators and destroyed habitats (referred to as “models with altered parameters”). Our analysis revealed that even with altered parameters, habitat destruction still mitigated the threats posed by global invasive predators to native predator–prey systems (refer to Figures S3–S6 in the Appendix S1). Furthermore, the counteractive effect of habitat destruction in the presence of multiple alien global predators remained consistent in models with altered parameters (refer to Figure S7 in the Appendix S1). Moreover, Figure S8 in the Appendix S1 shows that the conclusion regarding the stronger counteractive effect of low levels of habitat destruction occurring at a faster rate remained qualitatively robust, regardless of changes in the parameters of model. Based on these findings, we can confidently conclude that our observed results are robust and unaffected by variations in the parameters of the models.

## 4 DISCUSSION

**FIGURE 7:**
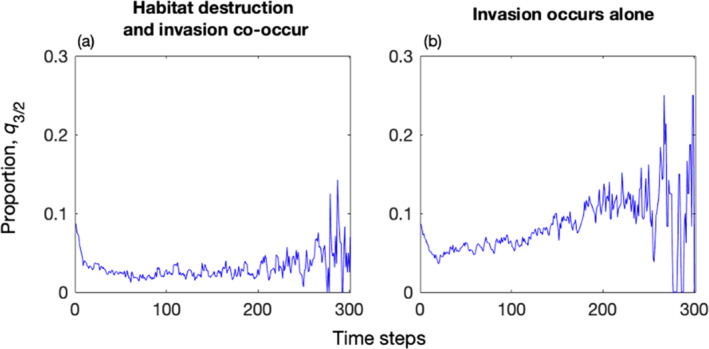
Habitat destruction prevents the encroachment of global invasive predators into areas surrounding native predators. For native systems containing invaders characterized by λ=1.67, η=1, b=0.8, and β=0.6, panels (a, b) illustrate the relationships between the proportions of global invasive predators in the nearest 4 neighbors of native predators and the corresponding time steps. The parameters are $m_{i}=0.2$, $c_{23}=0.15$, $n_{\text{alien}}=1000$, $D_{\mathrm{end}}=21\%$, $t_{0}=\text{step}\;6$, $t_{\eta }=6\;\text{steps}$, and $x_{\mu }=0.025$.

Habitat destruction can counterbalance the threats posed by global invasive predators to native predator–prey systems. This phenomenon is exemplified in Figure 7. In the absence of habitat destruction, invasion leads to a gradual increase in the proportion of invasive predators near native predators, rising from approximately 0.08 to over 0.15 over time and peaking at 0.25 (Figure 7b). However, when habitat destruction and predator invasion coincide, the proportion of invasive predators rapidly diminishes from approximately 0.08 to approximately 0.04 over time (Figure 7a). Therefore, habitat destruction effectively impedes the encroachment of invasive predators upon native predators. Native predators primarily contend with competitive pressure from invasive predators in the surrounding area, and habitat destruction markedly reduces the competitive influence exerted by invaders. Thus, habitat destruction can assist native systems in resisting invasion.

As analyzed in the previous paragraph, this assisting impact of habitat destruction on native system under global predator invasion likely create barriers that impede invasive species from threating native habitats. Therefore, this approach aligns with management practices that establish “barrier zones,” such as those implemented by the United States Forest Service to control the spread of gypsy moths (*Lymantria dispar*) (Sharov & Liebhold, 1998), employing eradication or suppression efforts to prevent or slow the expansion rate and thus mitigate the damage caused by gypsy moths to native species. In this sense, habitat destruction may serve as a tool for controlling alien species invasion (Bozzuto et al., 2021; Turner, 1989). However, this approach also entails risks. The analysis in Section 3.1 shows that habitat destruction can compensate the threats of invasive predators to the native system only when native predators present global predation strategy similar to invasive predators; otherwise, habitat destruction consistently accelerates the collapse of the native system under predator invasion. The analyses in Sections 3.2 and 3.3 show that, except for low levels of habitat destruction occurring at a faster rate, in conjunction with a substantial number of global invasive predators, the counteractive effects of habitat destruction and invasive predators on native systems are not obvious and may even be nonexistent.

Additionally, to our knowledge, no study has investigated the practical application of habitat destruction for controlling alien species invasion. This study, too, remains theoretical. Thus, the suggestions in this study do not imply that habitat destruction should be embraced as a universal management guideline for controlling alien species invasion. In fact, this research is guided by the recognition that habitat destruction constitutes a substantial and widespread threat to biodiversity (Crooks et al., 2017). We aim to mitigate this threat by transforming the impact of habitat destruction into something beneficial for biodiversity, compensating for the threats to native species from invaders, rather than resorting to the direct destruction of habitats to shield native species from predator invasion. In essence, we sought to explore whether and how to leverage habitat destruction, one of the most significant threats to biodiversity, to mitigate the impacts of predator invasion on native species.

Previous theoretical and experimental researches have focused on examining the impact of habitat destruction on biological invasions, including investigations into whether habitat destruction enhances (Wang et al., 2021) or inhibits (Alofs & Fowler, 2010; Bozzuto et al., 2021) invasions, as well as the role of habitat destruction in protecting weaker native competitors during invasions (Yang & Liu, 2023). In this study, we investigated a native predator–prey system that was simultaneously exposed to invasive predators and habitat destruction. The results indicate that when native predators have comparable predation capability to invaders, habitat destruction can counterbalance the threats posed by global invasive predators to native predator–prey system (Figures 3 and 4). However, if these conditions are not met, habitat destruction further exacerbates the collapse of the native system under predator invasion (Figures 3 and 4). Furthermore, our findings suggest that low levels of habitat destruction (Figure 6), rather than intermediate levels (Yang & Liu, 2023), are the most effective at mitigating the threats of global invasive predators to native predator–prey system. In contrast to the findings of previous works (Yang & Liu, 2023), we did not find evidence indicating that the timing of habitat destruction introduction or the spatial distribution of destroyed habitats contributed to the resistance of the native system to invasion.

Our findings support previous research suggesting that habitat destruction can impede biological invasions and protect native species from extinction (Alofs & Fowler, 2010; Bozzuto et al., 2021; Yang & Liu, 2023). Figure 3e,f, as well as Figure 4c,d, add further credibility to this idea. Additionally, Yang and Liu (2023) proposed that shortening the time between two habitat destruction events is more beneficial for assisting weaker native competitors during invasions. Our results in Figure 6c,d indicate that reducing the time interval between two habitat destruction events can also enhance the ability of the native predator–prey system to resist competition pressure from globally invasive predators. Therefore, our findings align with the conclusions reached by Yang and Liu (2023).

There are several intriguing avenues for further research. First, the complexity of the native food web plays a critical role in the response of native species to habitat destruction and invasion (Jiabu & Li, 2023). Expanding our predator–prey model to include a complex food web would allow us to examine how food web complexity affects responses when habitat destruction and invasion occur together. Second, since invasive species must rapidly adapt to new environmental conditions during invasion (Hänfling & Kollmann, 2002; Wang et al., 2023), investigating the evolutionary responses of both invasive and native species (Evans et al., 2022) could elucidate the impact of coevolution on invasion and persistence. Last, while the core conclusion of this study namely that habitat destruction may compensate threats to native predator–prey systems from global predator invasion, receives some mechanistic support (Figure 7) and is corroborated by other studies (Bozzuto et al., 2021), this conclusion holds true only under very specific conditions, as analyzed in the “Results” section and the second and third paragraphs of this “Discussion” section. Consequently, this core conclusion is susceptible to misinterpretation by readers and may even raise moral and ethical questions. Therefore, it is crucial not only to delve deeper into the conditions under which habitat destruction and global predator invasion compensate their respective threats to native species but also to integrate these findings with practical management measures. This deeper exploration will help us understand how to leverage the interaction of habitat destruction and predator invasion to mitigate threats to native species more effectively. Such efforts may facilitate better understanding and acceptance of our conclusions among readers. These areas will be the focal points of our future research endeavors.

## AUTHOR CONTRIBUTIONS

**Jing Zhang:** Methodology (equal); software (equal); writing – original draft (equal). **Linying Wang:** Methodology (equal); software (equal). **Yinghui Yang:** Methodology (equal); software (equal); supervision (equal). **Haoqi Liu:** Conceptualization (equal); project administration (lead); writing – original draft (equal); writing – review and editing (equal).

## CONFLICT OF INTEREST STATEMENT

The authors declare that they have no conflict of interest.

## CONSENT TO PARTICIPATE

All authors consent in participate in this manuscript.

## Supporting information


Appendix S1

## Data Availability

Data sharing not applicable to this article as no datasets were generated or analyzed during the current study.
