# Supplementary material for: Counteractive effects of predator invasion and habitat destruction on predator–prey systems
Source: Ecol Evol. 2024 Jul 4;14(7):e11646. doi: 10.1002/ece3.11646 (PMC11224505; doi:10.1002/ece3.11646)
Supplement: Supplementary file 1 — Appendix S1 [file ECE3-14-e11646-s001.docx]

We verified the robustness of our findings by examining the impact of variations in the parameters of the predator‒prey and habitat destruction models. Since the initial spatial distribution of invasive species and the process of habitat destruction significantly influence invasion processes and consequently affect our results, we tested the robustness of our findings to changes in these two parameters. To do this, we introduced a non‒random spatial distribution (centre invasion) to test the robustness of the results, as our main results were based on randomly distributed invasive species. Similarly, we also introduced a non‒random spatial process of habitat destruction (contagious habitat destruction) to test the robustness of the results, as we used random habitat destruction that corresponded to the random spatial process of habitat destruction in the main results. Subsequently, we compared the outcomes of the original models (referred to as "models with original parameters") to those of the models with altered parameters (referred to as "models with altered parameters").

To achieve this aim, in Section S1, we established the non-random spatial distribution (centre invasion). In Section S2, we established the non-random spatial process of habitat destruction (contagious habitat destruction). Finally, in Section S3, we compared the outcomes of the original models to those of the models with altered parameters.

**S1**. **Initial spatial distribution of invaders**


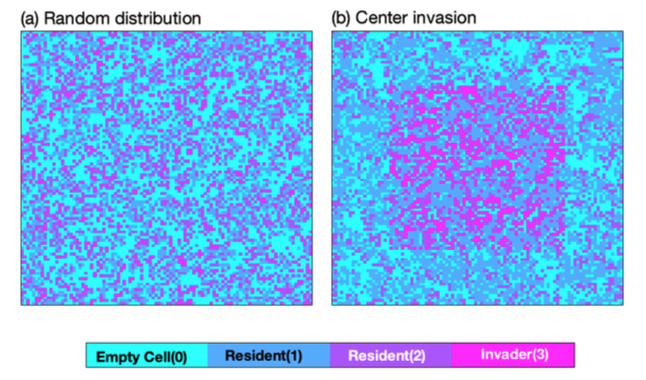


**Fig. S1**. Initial spatial distribution of global invasive predators.

We established central invasion in this section. Initially, we allocated 60% of the cells to native prey and 20% to predators, with the remaining cells remaining empty. Once the native system reached equilibrium, we identified $n_{\mathrm{alien}}$ empty cells in the centre of the landscape and introduced an invasive predator into each of these cells (refer to Fig. S1b). For information on the random distribution (as depicted in Fig. S1a), further details can be found in Section 2.1.

**S2**. **Non‒random habitat destruction**

We described contagious habitat destruction in this section. For contagious habitat destruction, if we used a Moore neighbourhood, all intact habitats should be divided into 9 groups, and in group $i (i=0,1,\ldots,8)$, each habitat has $i$ lost neighbouring habitats. Boakes et al. (2010) showed that a larger $i$ is associated with a greater probability that the habitat in group $i$ will be lost, i.e., $x_{i,t_{\zeta}}<x_{j,t_{\zeta}}$ ($i<j$), where $x_{i,t_{\zeta}}$ is the destruction probability of group $i$ at step $t_{\zeta}$. We assumed that the speed of random habitat destruction (refer to Section 2.2) was the same as the speed of contagious habitat destruction.

|  | $n_{\mu,t_{\zeta}}x_{\mu}=\sum_{i=0}^{8} n_{i,t_{\zeta}}x_{i,t_{\zeta}}$. | (S1) |
| --- | --- | --- |

Therefore, we could compare these two habitat destruction models, where $n_{\mu,t_{\zeta}}$ is the number of intact habitats at step $t_{\zeta}$ under random habitat destruction and $n_{i,t_{\zeta}}$ is the number of intact habitats with $i$ lost neighbouring habitats under contagious habitat destruction. Then, we introduced $ẟ_{0}$ and $r_{i}$ $(i=0,1,\ldots,8)$ and $0\leq r_{i}\leq r_{j}$ for $i<j$ (the values of $r_{i}$ are given in Fig. S2), and let $x_{i,t_{\zeta}}=ẟ_{0}r_{i}$ describe the degree to which habitat destruction is contagious. According to Equation (S1), we could obtain the values of $ẟ_{0}$ and therefore the values of $x_{i,t_{\zeta}}$. However, $x_{i,t_{\zeta}}$ may be larger than 1, which is unphysical since $x_{i,t_{\zeta}}$ represents probability. As $x_{i,t_{\zeta}}=ẟ_{0}r_{i}$ and $r_{i}\leq r_{j}$, for $i<j$, we could find the smallest index $i_{1}$ such that $x_{i,t_{\zeta}}>1$ for $i\geq i_{1}$ and $x_{j,t_{\zeta}}\leq1$ for $j<i_{1}$. Then, we let $x_{i,t_{\zeta}}=1$ for $i\geq i_{1}$; if $ẟ_{1}$ was introduced and $x_{i,t_{\zeta}}=ẟ_{1}r_{i}$, for $i<i_{1}$, we could obtain $ẟ_{1}=(n_{\mu,t_{\zeta}}x_{\mu}-\sum_{i=i_{1}}^{8} n_{i,t_{\zeta}})$/$\sum_{i=0}^{i_{1}-1} n_{i,t_{\zeta}}r_{i}$ and therefore the values of $x_{i,t_{\zeta}}$. If $x_{i,t_{\zeta}}>1$ persists, then the above step was repeated until $x_{i,t_{\zeta}}\leq1$ ($i=0,1,\ldots,8$). At this point, the contagious habitat destruction model was complete. We also obtained the proportion of cells destroyed at step $t_{\zeta}$ for the destruction of the contagious habitat:

|  | $D_{\mu,t_{\zeta}}=(\sum_{j=1}^{t_{\zeta}} \sum_{i=0}^{8} n_{i,j}x_{i,j})/10000$. | (S2) |
| --- | --- | --- |

where $10000$ is the total number of cells in the landscape (refer to Section 2.2). The results of the contagious habitat destruction model are given in Fig. S2.

**Fig. S2**. Examples of the contagious habitat destruction process. The blue areas are intact cells, and the white areas are destroyed cells. Parameters: $x_{\mu}=0.025$, $r_{0}=1$, $r_{1}=200$, $r_{2}=800$, $r_{3}=1,501$, $r_{4}=3,000$, $r_{5}=10,000$, $r_{6}=41,200$, $r_{7}=130,001$, $r_{8}=560,000$.

**S3**. **Robustness of results**

In Section S3.1, we assessed the robustness of the conclusion that the threats posed by global predation capability invasion and habitat destruction may have counteractive effects on the native system. In Section S3.2, we evaluated the robustness of the conclusion that habitat destruction and global predator invasion exert counteractive effects on the native system when faced with numerous alien global predators. In Section S3.3, we examined the robustness of the conclusion that habitat destruction and global predator invasion can more effectively mitigate their respective threats to the native system.

**S3.1 Findings regarding habitat destruction mitigating the threats posed by global invasive predators to native species are robust**

Figs. S3‒S6 demonstrate the persistence of the native system when habitat destruction and global predator invasion co‒occur, considering models with altered parameters. Our observations suggest that if the native predators possess similar global predation capability to invasive predators, the native systems have a greater chance of persistence when habitat destruction and invasion co‒occur compared to when only invasion occurs. In contrast, habitat destruction consistently accelerated the collapse of the native system under predator invasion in all the other scenarios. Consequently, conclusions regarding how habitat destruction compensates the threats posed by global invasive predators to the native system remain robust.


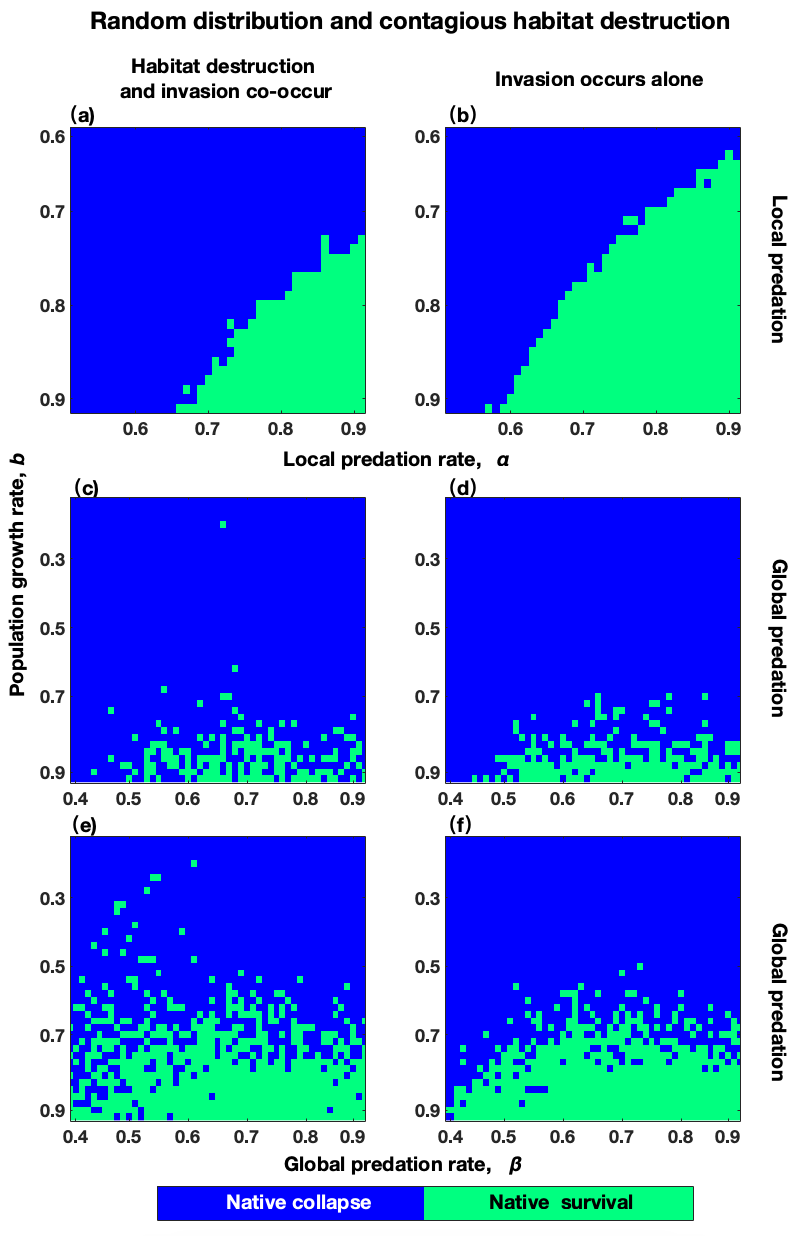


**Fig. S3**. Parameter space ($b$ and $\alpha$ for (a‒b) and $b$ and $\beta$ for (c‒f)) for the persistence of native systems when invaders possess competition advantages over the natives ($\lambda=1.67$ for (a‒f)) but invaders and natives have comparable predation ability ($\eta=$1 for (a‒b) and (e‒f)) or when the invaders hold predation advantages over the natives ($\eta=1.01$ for (c‒d)). The key parameters include $m_{i}=0.2$, $c_{23}=0.15$, $n_{\mathrm{alien}}=1000$, $D_{\mathrm{end}}=21\%$, $t_{0}=\mathrm{step}6$, and $t_{\eta}=6 \mathrm{steps}$; the remaining parameters are shown in Fig. S2.


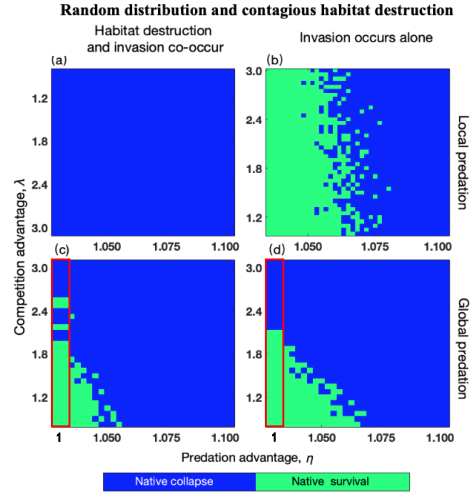


**Fig. S4**. Parameter space ($\lambda$ and $\eta$) for native system persistence when contagious habitat destruction and invasion co‒occur or invasion occurs alone. The horizontal axis for the red region in panels (c‒d) is labelled as 1. The key parameters include (a‒b) $\alpha=0.7$; (c‒d) $\beta=0.6$; $b=0.8$, $m_{i}=0.2$, $c_{23}=0.15$, $n_{\mathrm{alien}}=1000$, $D_{\mathrm{end}}=29\%$, $t_{0}=\mathrm{step}2$, and $t_{\eta}=2 \mathrm{steps}$; and the remaining parameters are shown in Fig. S2.


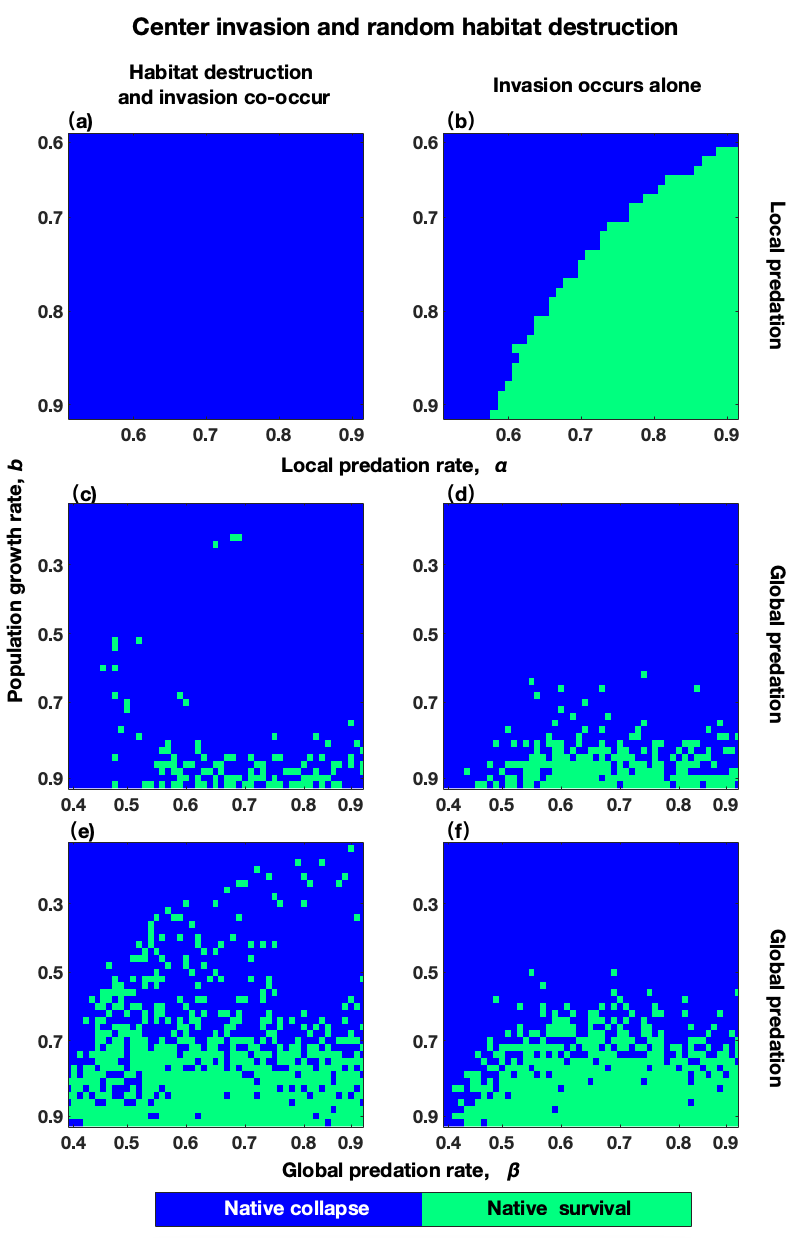


**Fig. S5**. Parameter space ($b$ and $\alpha$ for (a‒b) and $b$ and $\beta$ for (c‒f)) for the persistence of native systems when invaders possess competition advantages over the native ($\lambda=1.67$ for (a‒f)) but invaders and natives have comparable predation ability ($\eta=$1 for (a‒b) and (e‒f)) or when the invaders hold predation advantages over the natives ($\eta=1.01$ for (c‒d)). The key parameters include $m_{i}=0.2$, $c_{23}=0.15$, $n_{\mathrm{alien}}=1000$, $D_{\mathrm{end}}=29\%$, $t_{0}=\mathrm{step}2$, $t_{\eta}=2 \mathrm{steps}$, and $x_{\mu}=0.025$ for all panels.


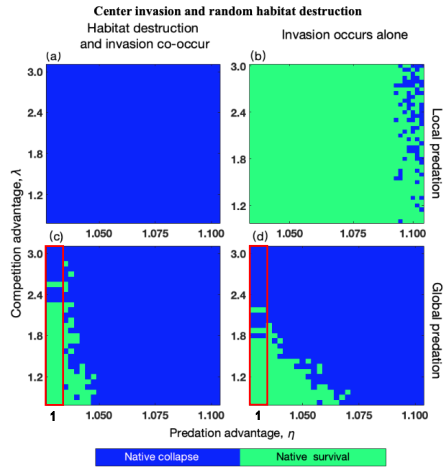


**Fig. S6**. Parameter space ($\lambda$ and $\eta$) for native system persistence when random habitat destruction and centre invasion co‒occur or centre invasion occurs alone. The horizontal axis for the red region in panels (c‒d) is labelled as 1. The key parameters include (a‒b) $\alpha=0.7$; (c‒d) $\beta=0.6$; $b=0.8$, $m_{i}=0.2$, $c_{23}=0.15$, $n_{\mathrm{alien}}=1000$, $D_{\mathrm{end}}=29\%$, $t_{0}=\mathrm{step}2$, $t_{\eta}=2 \mathrm{steps}$, and $x_{\mu}=0.025$ for all panels.

**S3.2 Conclusions regarding the counteractive effect of habitat destruction occurring only in the presence of multiple alien global predators are robust**

Fig. S7 shows whether habitat destruction can compensate the threats posed by global invasive predators to the native system when considering models with altered parameters. Qualitatively, habitat destruction and global invasive predators can counteractively affect the native system only when there is a significant number of alien predators, and these effects remain consistent across different parameter sets.

**
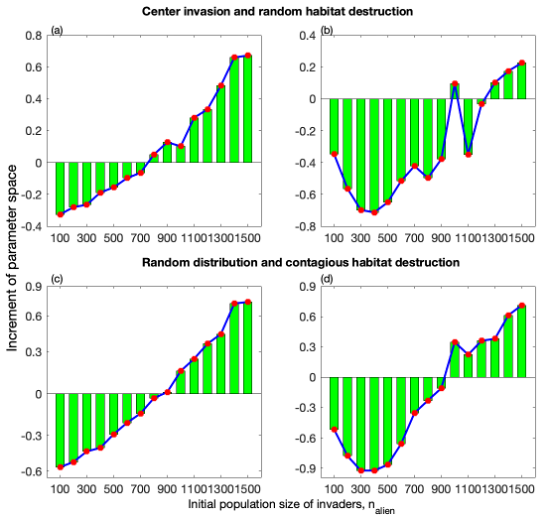
**

**Fig. S7.** Correlations between the initial population size of global invasive predators ($n_{\mathrm{alien}}$) and the expansion of parameter space ($b$ and $\beta$ for (a) and (c) and $\lambda$ and $\eta$ for (b) and (d)) for the persistence of the native system. Panels (a‒b) correspond to central invasion and random habitat destruction, and panels (c‒d) correspond to the random distribution of invaders and contagious habitat destruction. For systems without habitat destruction, the parameters are set as follows: in panels (a) and (c), $b=0.8$ and $\beta=0.6$, while in panels (b) and (d), $\lambda=1.67$ and $\eta=1$. When considering both scenarios with and without habitat destruction, the parameters are adjusted as follows: $\lambda=1.67$, $\eta=1$, $D_{\mathrm{end}}=21\%$, $t_{0}=\mathrm{step}6$, and $t_{\eta}=6 \mathrm{steps}$ for panels (a) and (c); $b=0.8$, $\beta=0.6$, $D_{\mathrm{end}}=29\%$, $t_{0}=\mathrm{step}2$, and $t_{\eta}=2 \mathrm{steps}$ for panels (b) and (d); and $m_{i}=0.2$, $c_{23}=0.15$, and $n_{\mathrm{alien}}=1000$ for all panels, with the remaining parameters shown in Fig. S2.


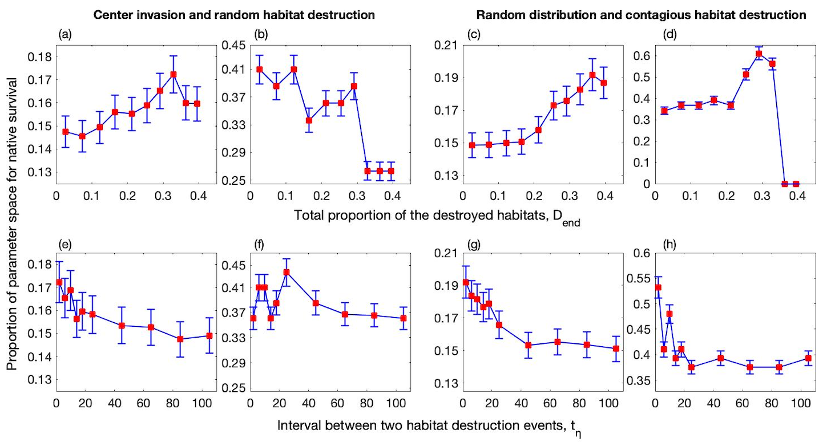


**Fig. S8.** Relationships between the proportion of parameter space ($b$ and $\beta$ for (a), (c), (e), and (g); $\lambda$ and $\eta$ for (b), (d), (f), and (h), in which the native systems can survive when habitat destruction and global predator invasion co‒occur and the total proportion of the destroyed habitats, $D_{\mathrm{end}}$, or the time interval between two habitat destruction events, $t_{\eta}$. Panels (a‒b) and (e‒f) correspond to central invasion and random habitat destruction, respectively, and panels (c‒d) and (g‒h) correspond to the random distribution of invaders and contagious habitat destruction, respectively. The parameters used for all panels are as follows: (a) and (c) $t_{\eta}=6 \mathrm{steps}$; (b) and (d) $t_{\eta}=2 \mathrm{steps}$; (e) and (g) $D_{\mathrm{end}}=21\%$; (f) and (h) $D_{\mathrm{end}}=29\%$; (a), (c), (e), and (g) $\lambda=1.67$, $\eta=1$, $t_{0}=\mathrm{step}6$; (b), (d), (f), and (h) $b=0.8$, $\beta=0.6$, $t_{0}=\mathrm{step}2$; $m_{i}=0.2$, $c_{23}=0.15$, and $n_{\mathrm{alien}}=1000$ for all panels, while the remaining parameters are shown in Fig. S2.

**S3.3 Conclusions regarding the stronger counteractive effect of low levels of habitat destruction occurring at a faster rate are robust**

Fig. S8 shows that for models with altered parameters, when the interval between two habitat destruction events is very short (indicating that habitat destruction occurred at a faster rate) or the level of habitat destruction is low, the native systems have a greater chance of persistence when habitat destruction and global predator invasion co‒occur. Hence, the conclusion that low levels of habitat destruction occur at a faster rate, along with global predator invasion, resulting in a stronger counteractive effect (as depicted in Fig. 6) remains qualitatively robust when considering models with altered parameters.

**References**

Boakes, E. H., Mace, G. M., McGowan, P. J. K., et al. (2010). Extreme contagion in global habitat clearance. Proceedings of the Royal Society B: Biological Sciences, 277, 1081‒1085.
